# Patient Characteristics Associated With Being Offered or Choosing Telephone vs Video Virtual Visits Among Medicare Beneficiaries

**DOI:** 10.1001/jamanetworkopen.2023.5242

**Published:** 2023-03-29

**Authors:** Ishani Ganguli, E. John Orav, Ruth Hailu, Joyce Lii, Meredith B. Rosenthal, Christine S. Ritchie, Ateev Mehrotra

**Affiliations:** 1 Division of General Internal Medicine and Primary Care, Brigham and Women's Hospital, Harvard Medical School, Boston, Massachusetts; 2 Division of General Internal Medicine and Primary Care, Brigham and Women's Hospital, Harvard T.H. Chan School of Public Health, Boston, Massachusetts; 3 Department of Health Care Policy, Harvard Medical School, Boston, Massachusetts; 4 Department of Health Care Policy and Management, Harvard T.H. Chan School of Public Health, Boston, Massachusetts; 5 Mongan Institute and the Division of Palliative Care and Geriatric Medicine, Massachusetts General Hospital, Harvard Medical School, Boston, Massachusetts

## Abstract

**Importance:**

After the rapid expansion of telemedicine during the COVID-19 pandemic, there is debate about the role and reimbursement of telephone vs video visits. Missing is an understanding of what type of virtual visits clinicians may offer or patients may choose when given the option.

**Objective:**

To evaluate characteristics of Medicare beneficiaries associated with practices and clinicians offering telephone visits only and patients receiving telephone visits only, when both telephone and video were available.

**Design, Setting, and Participants:**

This survey study used 2019-2020 nationally representative Medicare Current Beneficiary Survey data. Participants included community-dwelling Medicare beneficiaries with a usual source of medical care who attended a practice offering telemedicine. Data were analyzed from May 3 to August 23, 2022.

**Main Outcomes and Measures:**

Multivariable regression analysis was used to identify patient sociodemographic (age, sex, race, ethnicity, educational level, income, English proficiency, housing type, and number living at home), clinical (dementia, mental illness, self-rated health, hearing impairment, and vision impairment), and technology (technology access and prior use of video visits) factors associated with respondents’ report of (1) practices offering telephone virtual visits only, (2) being offered telephone visits only when both video and telephone visits were available, and (3) receiving telephone visits only when both video and telephone visits were offered.

**Results:**

Of 4691 respondents (representing 27 887 642 Medicare beneficiaries; mean [SD] age, 71.3[8.1] years; 55.0% female) reporting that their practice offered telemedicine, 1234 (23.3% weighted) reported that their practices offered telephone virtual visits only; factors associated with being in a practice offering telephone only included older age (adjusted odds ratio [aOR], 1.62 [95% CI, 1.10-2.39] for those aged ≥85 years vs 18-64 years), male sex (aOR, 1.36 [95% CI, 1.12-1.64]), Hispanic ethnicity (aOR, 1.41 [95% CI, 1.03-1.95]), lower income (aOR, 1.89 [95% CI, 1.43-2.49] for those with income ≤100% vs >200% of the federal poverty level), poor self-rated health (aOR, 1.25 [95% CI, 1.01-1.56]), and less technology access (aOR, 2.05 [95% CI, 1.61-2.60] for those with low vs high access). Of the 1593 patients in practices offering both video and telephone visits, 297 (16.7% weighted) were themselves offered telephone visits only; factors associated with being offered telephone only included Hispanic ethnicity (aOR, 1.96 [95% CI, 1.13-3.41]), limited English proficiency (aOR, 3.05 [95% CI, 1.28-7.31]), and less technology access (aOR, 1.68 [95% CI, 1.00-2.81] for those with low vs high access). Finally, of the 711 respondents who were themselves offered both video and telephone visits, 304 (43.1% weighted) had a telephone visit; factors associated with receiving telephone visits only were older age (aOR, 2.68 [95% CI, 1.21-5.92] for those aged 75-84 years vs 18-64 years) and less technology access (aOR, 2.65 [95% CI, 1.12-6.25] for those with moderate vs high access]). Among those who used video calls in other settings and were offered a choice, 122 (28.5%, weighted) chose telephone visits.

**Conclusions and Relevance:**

In this survey study of Medicare beneficiaries, respondents often reported being offered or choosing telephone visits even when video visits were available. Study findings suggest that policy makers and clinical leaders should support the use of telephone visits to the extent that telephone is appropriate, while addressing both practice-level and patient-level barriers to video visits.

## Key Points

**Question:**

What patient characteristics are associated with being offered or choosing telephone vs video visits?

**Findings:**

In this survey study of 4691 Medicare beneficiaries (representing 27 887 642 Medicare beneficiaries), 17% receiving care from practices offering both video and telephone visits reported that they were personally offered telephone visits only; 43% of those who were personally offered both video and telephone visits chose telephone visits. Being offered and choosing telephone visits were associated with less technology access and lack of video experience; those with Hispanic ethnicity or limited English proficiency were more likely to be offered telephone visits but not more likely to choose them.

**Meaning:**

This study found that many patients reported choosing telephone visits when given the option, suggesting the need to support telephone visits when appropriate while addressing multilevel barriers to video use (eg, clinic infrastructure and interpreter availability).

## Teaser

This survey study of Medicare beneficiaries evaluates patient characteristics associated with practices and clinicians offering telephone visits only and patients receiving telephone visits only, when both telephone and video were available.

## Introduction

After the rapid expansion of virtual visits via both video and telephone during the COVID-19 pandemic,^1,2,3,4^ there is ongoing debate about the role of telephone visits and, specifically, whether Medicare and other payers should continue to reimburse clinicians for them. On the one hand, compared with video visits, telephone visits do not allow for visual patient examination or eye contact and may be more prone to overuse and fraud.^5,6^ On the other hand, for some patients and in some scenarios, telephone visits may be clinically equivalent to video and may represent the only option (eg, due to lack of internet or digital devices) or the preferred option (eg, due to ease of use or desire for privacy in a crowded home environment).^5^

Based on evidence to date, policy makers and clinical leaders have largely assumed that the use of telephone visits is due to patients lacking access to video technology (ie, the “digital divide” that is mediated by factors such as structural racism).^1,5^ This includes evidence, predominantly from single-site studies of clinics and health systems, of differences by sociodemographic characteristics in the receipt of video vs telephone visits. Specifically, these studies show that telephone visits are more often used by patients who are older,^7,8,9,10,11,12,13,14,15,16^ are of minoritized race^7,8,9,10,11,12,13,14,15,17^ or Hispanic ethnicity,^8,9,16^ have limited English proficiency,^7,9,13,17^ are enrolled in Medicaid or otherwise identified as having low incomes,^8,9^ have less education,^10^ live in rural areas,^7^ or live in areas with less broadband internet access.^9^ What has received less attention is the role that clinicians play in driving these differences. Some practices or clinicians may not offer video visits or may only offer video visits to a subset of their patients; 1 study of a large health system found that variation in visit modality was explained more by practices and clinicians than by patients.^9^ Similarly, the role of patient choice in telephone visits remains unclear—for example, how often do patients choose telephone visits when given both options, and what might drive this choice?

Understanding how the use of telephone visits may be influenced by practice availability, clinician offerings, and patient choice would inform policies and implementation strategies to ensure that patients have access to telemedicine that meets their needs, whether these visits are by video or telephone. To this end, we used nationally representative 2019-2020 Medicare Current Beneficiary Survey (MCBS) data to assess patient sociodemographic, clinical, and technology factors associated with the decision to offer or choose video vs telephone visits at each stage of the decision-making process—from the practice’s decision to offer telephone or video visits, in general, to a clinical team member’s decision to offer a telephone vs video visit to a given patient, and to the final choice of modality.

## Methods

### Data Source

The MCBS is a longitudinal survey with a rotating panel design of a representative national sample of traditional Medicare and Medicare Advantage beneficiaries. This survey is conducted in English and Spanish and enriched for Hispanic and older adult populations. We used the 2019 survey and the COVID-19 fall 2020 supplement (administered from October 5 to November 15, 2020) to sample beneficiaries who were continuously enrolled in Medicare from the start of 2020 through fall 2020. Responses to the 2 surveys are linked by an encrypted identifier. The Mass General Brigham institutional review board considered this study exempt from review and from the requirement for informed consent per 45 CFR 46.101(b). The study followed the Strengthening the Reporting of Observational Studies in Epidemiology (STROBE) reporting guideline.

### Study Population

We limited the sample to adult beneficiaries who were community dwelling, continuously enrolled in 2019, responded to the COVID-19 fall supplement, had a usual source of medical care, and responded to the question “Does your usual provider offer telephone or video appointments, so that you don’t need to physically visit their office or facility?”

### Study Outcomes

We used a series of survey items about telemedicine in the COVID-19 supplement to create 3 groups of respondents (Figure). Group 1 was composed of respondents at practices offering telemedicine (defined by a “yes” response to the aforementioned question) and a nonmissing response to the question “Do they offer telephone appointments, video appointments, or both?” For group 1, the outcome of interest was the usual practice offering telephone visits only (vs video visits only or both).

**Figure.**
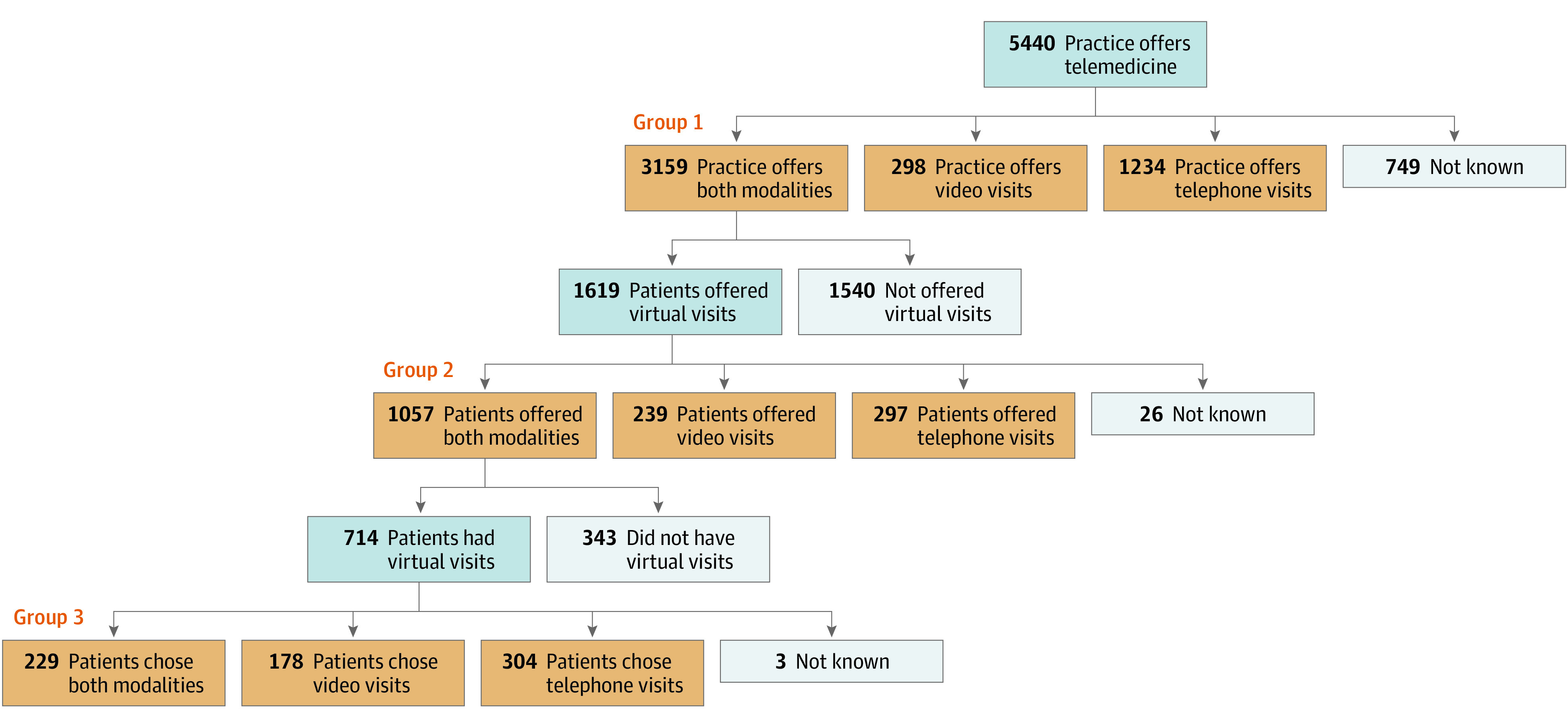
Flowchart of Respondents Reporting on Video and Telephone Visit Modalities

Group 2 was the subset of group 1 composed of respondents at practices offering both telephone and video visits (defined by a “both” response to “Do they offer telephone appointments, video appointments, or both?”) who had been recently offered a virtual visit (defined by a “yes” response to “Since July 1, 2020, did your usual provider offer you a telephone or video appointment to replace a regularly scheduled appointment?” and a nonmissing response to “Did they offer telephone appointments, video appointments, or both?”). For group 2, the outcome of interest was the patient being offered telephone visits only (vs video visits only or both).

Group 3 was the subset of group 2 composed of respondents who were offered both telephone and video visits since July 1, 2020 (defined by a “both” response to “Did they offer telephone appointments, video appointments, or both?”) and had received a virtual visit (defined by a “yes” response to “Since July 1, 2020, have you had an appointment with a doctor or other health professional by telephone or video?” and a nonmissing response to “Was it a telephone appointment, video appointment, or both?”). For group 3, the outcome of interest was the patient receiving a telephone visit only (vs a video visit only or both).

### Covariates

Based on literature review and clinical experience, we chose sociodemographic, clinical, and technology variables that may be associated with patient or clinician propensity to choose a given telemedicine modality.^5^

#### Sociodemographic Variables

Sociodemographic variables collected included age (18-64, 65-74, 75-84, or ≥85 years), sex (male or female), race (self-reported; Black, White, or other [including American Indian or Alaska Native, Asian, Native Hawaiian or Pacific Islander, or >1 race]), Hispanic ethnicity (yes or no), educational level (did not graduate high school, high school or some college, or college or above), income (≤100% federal poverty level [FPL], >100% to ≤200% FPL, or >200% FPL), limited English proficiency (speaks English not well or not at all vs very well or well), housing type (apartment or condominium, house [including single-family home, 2-family home, rowhouse, or townhouse], mobile home or trailer), and number of people living in home (1, 2, or ≥3).

#### Clinical Variables

Clinical variables collected included dementia (Alzheimer disease or any type of dementia other than Alzheimer disease), mental illness (depression or a mental or psychiatric disorder other than depression), poor self-rated health (fair or poor vs excellent, very good, or good), hearing impairment (yes or no), and vision impairment (yes or no).

#### Technology Variables

There were 2 technology variables. The first, technology access group (created by the authors based on 5 survey items in the COVID-19 supplement),^18^ was defined as the following: high access (has internet access and has a smartphone or tablet); moderate access (has a smartphone but no internet access or has internet access and a computer but no smartphone or tablet); or low access (does not have a smartphone, internet access, or a computer). The second variable was use of internet-based video or voice calls (yes or no answer to the question “Since July 1, 2020, have you participated in video or voice calls or conferencing over the internet, such as with Zoom, Skype, or FaceTime?”).

### Statistical Analysis

Statistical analysis was conducted from May 3 to August 23, 2022. For each of our 3 outcomes, we calculated the proportion of eligible survey respondents who had the outcome. We then used multivariable logistic regression to assess sociodemographic, clinical, and technology factors associated with choice of telephone visit at each stage: (1) what the practice offered (group 1), (2) what the respondent was personally offered (group 2), and (3) what the respondent received (group 3). To explore the third stage further, we performed an additional analysis; because the number of people in one’s household may differentially motivate one’s choice of telephone vs video visit for residents of more vs less restrictive housing situations (eg, due to the desire for privacy), we added an interaction term for number of people in the household and housing type in this model.

We applied MCBS sampling weights throughout to construct national estimates and used balanced repeated replication weights for variance estimation.^19,20^ We considered 2-tailed *P* values significant at *P* < .05 and addressed missingness using complete case analysis. To evaluate differences between respondents and nonrespondents, we computed standardized mean differences. Data were analyzed using SAS, version 9.4 (SAS Institute Inc).

## Results

We assessed results from 5440 respondents (representing 32 143 458 Medicare beneficiaries; mean [SD] age, 71.5 [8.2] years; 55.5% women) who reported that their practice offered telehealth (Figure). Compared with nonrespondents for outcomes 1 and 2 (what practices offered and what a respondent was personally offered, respectively), respondents were younger and more likely to be Black, to be Hispanic, to have lower educational level, to have lower income status, to have limited English proficiency, or to report mental illness (eTable in Supplement 1).

Of 4691 respondents (representing 27 887 642 Medicare beneficiaries; mean [SD] age, 71.3[8.1] years; 55.0% female) who responded regarding modalities offered (group 1), 1234 (23.3% weighted) reported that their practices offered telephone only, 298 (6.6% weighted) reported that their practices offered video only, and 3159 (70.1% weighted) reported that their practices offered both (Table 1). In multivariable analyses, factors associated with being in a practice offering telephone only included older age (adjusted odds ratio [aOR], 1.62 [95% CI, 1.10-2.39] for those aged ≥85 years), male sex (aOR, 1.36 [95% CI, 1.12-1.64]), Hispanic ethnicity (aOR, 1.41 [95% CI, 1.03-1.95]), lower income (aOR, 1.89 [95% CI, 1.43-2.49] for those with income ≤100% of the FPL), poor self-rated health (aOR, 1.25 [95% CI, 1.01-1.56]), and having moderate or low technology access (moderate: aOR, 1.48 [95% CI, 1.12-1.95]; low: aOR, 2.05 [95% CI, 1.61-2.60]). Respondents who had used video calls in other settings (eg, with Zoom, Skype, or FaceTime) and those with dementia were less likely to report that their practice offered telephone visits only.

**Table 1.** Sociodemographic, Clinical, and Technology Factors Associated With Patients Reporting That Their Practice Offered Telephone Visits Only (Group 1)^a^

Characteristic	Patients, No. (%)			Adjusted OR of practice offering telephone visits only (95% CI)^b^
	Telephone visits (n = 1234)	Video visits (n = 298)	Both (n = 3159)	
Age, y				
18-64	177 (24.4)	61 (7.9)	532 (67.7)	1 [Reference]
65-74	365 (18.9)	113 (6.7)	1289 (74.4)	0.98 (0.72-1.35)
75-84	467 (28.2)	94 (6.1)	1000 (65.7)	1.42 (1.00-2.01)
≥85	225 (35.8)	30 (5.0)	338 (59.2)	1.62 (1.10-2.39)
Sex				
Male	596 (25.6)	124 (5.7)	1396 (68.6)	1.36 (1.12-1.64)
Female	638 (21.4)	174 (7.3)	1763 (71.3)	1 [Reference]
Race^c^				
Black	149 (27.9)	18 (3.9)	306 (68.2)	1.07 (0.80-1.44)
White	974 (22.4)	264 (7.1)	2580 (70.5)	1 [Reference]
Other	69 (25.6)	9 (6.3)	189 (68.1)	1.00 (0.70-1.42)
Hispanic ethnicity^d^				
Yes	236 (38.4)	20 (3.7)	201 (57.9)	1.41 (1.03-1.95)
No	996 (21.8)	277 (6.9)	2839 (71.3)	1 [Reference]
Educational level^e^				
Did not graduate high school	270 (40.0)	30 (4.4)	377 (55.6)	1.13 (0.83-1.52)
High school or some college	592 (24.0)	130 (5.8)	1519 (70.2)	1.02 (0.82-1.27)
College or above	366 (18.0)	135 (8.0)	1257 (73.9)	1 [Reference]
Income, FPL				
≤100%	304 (41.1)	37 (4.8)	439 (54.1)	1.89 (1.43-2.49)
>100% to ≤200%	342 (26.4)	71 (5.8)	777 (67.8)	1.13 (0.93-1.38)
>200%	588 (18.8)	190 (7.3)	1943 (73.9)	1 [Reference]
Limited English proficiency^f^				
Yes	167 (50.1)	12 (3.3)	149 (46.6)	1.45 (0.94-2.21)
No	1067 (21.9)	286 (6.8)	3005 (71.3)	1 [Reference]
Housing type^g^				
Apartment or condominium	234 (25.6)	53 (6.8)	525 (67.6)	1.01 (0.77-1.32)
Trailer	95 (28.7)	18 (7.3)	176 (64.0)	1.17 (0.89-1.54)
House	904 (22.5)	226 (6.5)	2451 (71.0)	1 [Reference]
No. of people in home				
1	357 (22.9)	84 (7.2)	837 (70.0)	1 [Reference]
2	593 (22.6)	143 (6.4)	1630 (71.0)	1.14 (0.94-1.39)
≥3	284 (25.6)	71 (6.5)	692 (67.9)	1.03 (0.79-1.33)
Dementia^h^				
Yes	50 (25.9)	12 (5.9)	126 (68.2)	0.57 (0.37-0.89)
No	1184 (23.2)	286 (6.6)	3030 (70.1)	1 [Reference]
Mental illness^i^				
Yes	344 (22.0)	107 (7.1)	1008 (71.0)	0.87 (0.71-1.06)
No	886 (23.8)	191 (6.4)	2147 (69.7)	1 [Reference]
Poor self-rated health^j^				
Yes	311 (29.2)	56 (5.4)	674 (65.4)	1.25 (1.01-1.56)
No	921 (21.7)	241 (7.0)	2479 (71.4)	1 [Reference]
Hearing impairment^k^				
Yes	224 (25.4)	35 (4.6)	529 (70.0)	0.99 (0.81-1.21)
No	1009 (22.9)	263 (7.0)	2627 (70.1)	1 [Reference]
Vision impairment^l^				
Yes	118 (27.4)	29 (6.4)	253 (66.1)	0.92 (0.67-1.28)
No	1114 (23.0)	269 (6.6)	2900 (70.4)	1 [Reference]
Technology access				
High	663 (18.4)	234 (7.3)	2405 (74.3)	1 [Reference]
Moderate	181 (31.1)	25 (5.4)	343 (63.5)	1.48 (1.12-1.95)
Low	390 (45.2)	39 (3.6)	411 (51.2)	2.05 (1.61-2.60)
Use of internet-based video or voice calls^m^				
Yes	360 (15.6)	183 (8.9)	1568 (75.5)	0.60 (0.50-0.73)
No	871 (30.7)	113 (4.4)	1585 (64.8)	1 [Reference]

Abbreviations: FPL, federal poverty level; OR, odds ratio.

^a^ Unweighted numbers and weighted row percentages of responses to each survey item among respondents in group 1 are presented (Figure).

^b^ The multivariable model included all listed covariates. In the model, missing values were either categorized as no (for ethnicity, dementia, mental illness, hearing impairment, and vision impairment) or included using an indicator variable.

^c^ Other race includes American Indian or Alaska Native, Asian, Native Hawaiian or Pacific Islander, and more than 1 race. A total of 133 respondents answered “do not know” or refused to answer regarding race.

^d^ A total of 122 respondents answered “do not know” or refused to answer regarding ethnicity.

^e^ A total of 15 respondents answered “do not know” or refused to answer regarding educational level.

^f^ A total of 5 respondents answered “do not know” or refused to answer regarding limited English proficiency.

^g^ A total of 9 respondents answered “do not know” or refused to answer regarding housing type.

^h^ A total of 3 respondents answered “do not know” or refused to answer regarding dementia.

^i^ A total of 8 respondents answered “do not know” or refused to answer regarding mental illness.

^j^ A total of 9 respondents answered “do not know” or refused to answer regarding poor self-rated health.

^k^ A total of 4 respondents answered “do not know” or refused to answer regarding hearing impairment.

^l^ A total of 8 respondents answered “do not know” or refused to answer regarding vision impairment.

^m^ A total of 11 respondents answered “do not know” or refused to answer regarding use of internet-based video or voice calls.

Of the 1593 respondents who reported that their practices offered both and were offered virtual visits (group 2), 297 (16.7% weighted) were personally offered telephone visits only (Table 2). In multivariable analyses, factors associated with being offered telephone visits only included Hispanic ethnicity (aOR, 1.96 [95% CI, 1.13-3.41]), limited English proficiency (aOR, 3.05 [95% CI, 1.28-7.31]), having more than 1 person in one’s home (vs 1; aOR, 1.57 [95% CI, 1.05-2.35] for 2 people), and having moderate or low technology access (moderate: aOR, 1.44 [95% CI, 0.94-2.22]; low: aOR, 1.68 [95% CI, 1.00-2.81]). Respondents who used video calls in other settings were less likely to report that they were offered telephone visits only.

**Table 2.** Sociodemographic, Clinical, and Technology Factors Associated With Respondents Reporting That They Were Offered Telephone Visits Only (Group 2)^a^

Characteristic	Respondents, No. (%)			Adjusted OR of being offered telephone visits only (95% CI)^b^
	Telephone visits (n = 297)	Video visits (n = 239)	Both (n = 1057)	
Age, y				
18-64	42 (12.5)	46 (14.3)	208 (73.2)	1 [Reference]
65-74	112 (16.3)	98 (18.1)	419 (65.6)	1.32 (0.70-2.46)
75-84	107 (19.0)	71 (14.2)	331 (66.9)	1.43 (0.71-2.89)
≥85	36 (21.7)	24 (15.1)	99 (63.2)	1.26 (0.58-2.78)
Sex				
Male	139 (17.2)	113 (16.8)	481 (66.0)	0.98 (0.69-1.39)
Female	158 (16.4)	126 (16.0)	576 (67.7)	1 [Reference]
Race^c^				
Black	32 (15.1)	23 (21.1)	101 (63.8)	0.95 (0.53-1.71)
White	235 (17.1)	200 (16.5)	835 (66.4)	1 [Reference]
Other	21 (16.1)	13 (10.2)	76 (73.8)	0.88 (0.49-1.57)
Hispanic ethnicity^d^				
Yes	45 (25.8)	17 (8.7)	108 (65.5)	1.96 (1.13-3.41)
No	252 (15.9)	222 (17.2)	942 (66.9)	1 [Reference]
Educational level^e^				
Did not graduate high school	48 (23.1)	20 (9.9)	128 (67.0)	1.06 (0.56-2.01)
High school or some college	160 (19.4)	105 (15.0)	500 (65.7)	1.32 (0.93-1.88)
College or above	88 (12.7)	114 (19.1)	429 (68.1)	1 [Reference]
Income, FPL				
≤100%	42 (17.1)	21 (10.7)	161 (72.2)	0.58 (0.30-1.11)
>100% to ≤200%	91 (19.8)	59 (15.3)	274 (64.9)	1.00 (0.66-1.50)
>200%	164 (15.5)	159 (17.6)	622 (66.9)	1 [Reference]
Limited English proficiency^f^				
Yes	26 (37.6)	8 (9.7)	40 (52.7)	3.05 (1.28-7.31)
No	271 (16.0)	231 (16.6)	1016 (67.4)	1 [Reference]
Housing type^g^				
Apartment or condominium	53 (17.4)	41 (16.2)	179 (66.4)	1.15 (0.75-1.75)
Trailer	26 (24.3)	11 (12.0)	59 (63.7)	1.78 (0.87-3.64)
House	217 (6.0)	187 (16.8)	817 (67.2)	1 [Reference]
No. of people in home				
1	77 (14.7)	62 (17.4)	286 (67.9)	1 [Reference]
2	157 (18.6)	130 (16.9)	517 (64.5)	1.57 (1.05-2.35)
≥3	63 (14.9)	47 (13.9)	254 (71.2)	1.03 (0.64-1.66)
Dementia^h^				
Yes	9 (16.2)	8 (13.7)	44 (70.2)	0.54 (0.18-1.59)
No	288 (16.8)	231 (16.5)	1011 (66.7)	1 [Reference]
Mental illness^i^				
Yes	103 (17.2)	89 (16.6)	374 (66.2)	1.18 (0.88-1.60)
No	193 (16.5)	150 (16.3)	681 (67.2)	1 [Reference]
Poor self-rated health^j^				
Yes	73 (16.9)	50 (15.9)	258 (67.3)	0.93 (0.61-1.43)
No	224 (16.7)	189 (16.5)	797 (66.7)	1 [Reference]
Hearing impairment^k^				
Yes	59 (18.8)	38 (15.0)	172 (66.2)	1.13 (0.76-1.69)
No	238 (16.4)	201 (16.7)	883 (67.0)	1 [Reference]
Vision impairment^l^				
Yes	26 (16.6)	17 (11.2)	88 (72.3)	0.95 (0.51-1.75)
No	270 (16.7)	222 (16.8)	966 (66.4)	1 [Reference]
Technology access^m^				
High	192 (14.2)	192 (16.6)	835 (69.2)	1 [Reference]
Moderate	44 (24.5)	23 (15.2)	99 (60.4)	1.44 (0.94-2.22)
Low	61 (28.4)	23 (15.8)	123 (55.9)	1.68 (1.00-2.81)
Use of internet-based video or voice calls^n^				
Yes	109 (11.3)	178 (21.3)	590 (67.5)	0.48 (0.35-0.66)
No	188 (24.1)	60 (9.8)	464 (66.1)	1 [Reference]

Abbreviations: FPL, federal poverty level; OR, odds ratio.

^a^ Unweighted numbers and weighted row percentages of responses to each survey item among respondents in group 2 are presented (Figure).

^b^ The multivariable model included all listed covariates. In the model, missing values were either categorized as no (for ethnicity, dementia, mental illness, hearing impairment, and vision impairment) or included using an indicator variable.

^c^ Other race includes American Indian or Alaska Native, Asian, Native Hawaiian or Pacific Islander, and more than 1 race. A total of 57 respondents answered “do not know” or refused to answer regarding race.

^d^ A total of 7 respondents answered “do not know” or refused to answer regarding ethnicity.

^e^ A total of 1 respondent answered “do not know” or refused to answer regarding educational level.

^f^ A total of 1 respondent answered “do not know” or refused to answer regarding limited English proficiency.

^g^ A total of 3 respondents answered “do not know” or refused to answer regarding housing type.

^h^ A total of 2 respondents answered “do not know” or refused to answer regarding dementia.

^i^ A total of 3 respondents answered “do not know” or refused to answer regarding mental illness.

^j^ A total of 2 respondents answered “do not know” or refused to answer regarding poor self-rated health.

^k^ A total of 2 respondents answered “do not know” or refused to answer regarding hearing impairment.

^l^ A total of 4 respondents answered “do not know” or refused to answer regarding vision impairment.

^m^ A total of 1 respondent answered “do not know” or refused to answer regarding technology access.

^n^ A total of 4 respondents answered “do not know” or refused to answer regarding use of internet-based video or voice calls.

Finally, of the 711 respondents who reported that they were offered both telephone and video visits and had a telemedicine visit (group 3), 304 (43.1% weighted) reported receiving a telephone visit (Table 3). In multivariable analyses, factors associated with receiving a telephone visit only were older age (specifically, 75-84 years vs 18-64 years; aOR, 2.68 [95% CI, 1.21-5.92]), having moderate (vs high) technology access (aOR, 2.65 [95% CI, 1.12-6.25]), and higher educational level (college or above vs not graduated high school; aOR for those who did not graduate high school, 0.30 [95% CI, 0.13-0.66]). Once again, respondents who used video calls in other settings were less likely to report that they received telephone visits only, although 122 of these patients (28.5%, weighted) still chose telephone visits. When we added an interaction term between the number of people in the household and housing type to this model, the interaction was not significant.

**Table 3.** Sociodemographic, Clinical, and Technology Factors Associated With Respondents Reporting That They Received Telephone Visits Only (Group 3)^a^

Characteristic	Respondents, No. (%)			Adjusted OR of receiving telephone visits only (95% CI)^b^
	Telephone visits (n = 304)	Video visits (n = 178)No. (%)	Both (n = 229)	
Age, y				
18-64	48 (32.9)	38 (25.2)	71 (41.9)	1 [Reference]
65-74	105 (39.7)	74 (26.6)	91 (33.7)	1.45 (0.74-2.84)
75-84	114 (56.5)	48 (22.9)	48 (20.6)	2.68 (1.21-5.92)
≥85	37 (50.8)	18 (24.6)	19 (24.6)	1.82 (0.62-5.36)
Sex				
Male	149 (44.2)	83 (24.9)	104 (30.9)	1.05 (0.68-1.62)
Female	155 (42.1)	95 (25.7)	125 (32.3)	1 [Reference]
Race^c^				
Black	36 (45.2)	16 (16.4)	28 (38.5)	1.42 (0.62-3.26)
White	239 (42.8)	146 (27.3)	174 (29.9)	1 [Reference]
Other	19 (45.2)	12 (23.4)	15 (31.4)	1.26 (0.45-3.30)
Hispanic ethnicity^d^				
Yes	28 (33.5)	18 (22.6)	39 (43.9)	0.78 (0.39-1.55)
No	273 (44.0)	159 (25.7)	189 (30.3)	1 [Reference]
Educational level				
Did not graduate high school	29 (31.5)	19 (20.2)	37 (48.2)	0.30 (0.13-0.66)
High school or some college	153 (48.2)	65 (19.5)	102 (32.3)	0.76 (0.49-1.18)
College or above	122 (40.9)	94 (31.4)	90 (27.8)	1 [Reference]
Income, FPL				
≤100%	49 (45.2)	24 (19.3)	47 (35.5)	2.31 (0.99-5.40)
>100% to ≤200%	87 (50.8)	35 (18.0)	61 (31.2)	1.52 (0.89-2.62)
>200%	168 (39.7)	119 (29.3)	121 (31.0)	1 [Reference]
Limited English proficiency^e^				
Yes	9 (26.8)	2 (9.6)	15 (63.6)	0.59 (0.13-2.71)
No	295 (43.5)	176 (25.7)	213 (30.8)	1 [Reference]
Housing type				
Apartment or condominium	55 (44.5)	36 (28.4)	44 (27.1)	0.95 (0.56-1.63)
Trailer	22 (51.9)	8 (23.8)	10 (24.3)	1.40 (0.48-4.13)
House	227 (42.1)	134 (24.7)	175 (33.2)	1 [Reference]
No. of people in home				
1	98 (50.6)	42 (21.3)	63 (28.1)	1 [Reference]
2	135 (40.2)	93 (28.3)	94 (31.5)	0.78 (0.51-1.20)
≥3	71 (39.9)	43 (24.5)	72 (35.6)	0.95 (0.56-1.63)
Dementia^f^				
Yes	10 (37.7)	10 (35.4)	8 (26.9)	0.47 (0.11-1.95)
No	294 (43.4)	168 (25.1)	220 (31.5)	1 [Reference]
Mental illness^g^				
Yes	102 (41.5)	60 (23.7)	93 (34.8)	1.20 (0.75-1.93)
No	202 (44.2)	118 (26.3)	135 (29.5)	1 [Reference]
Poor self-rated health^h^				
Yes	65 (37.7)	48 (24.4)	78 (38.0)	0.63 (0.38-1.06)
No	238 (45.2)	130 (25.7)	150 (29.0)	1 [Reference]
Hearing impairment^i^				
Yes	54 (40.5)	28 (22.9)	39 (36.6)	1.04 (0.61-1.78)
No	250 (43.8)	150 (25.9)	188 (30.3)	[Reference]
Vision impairment^j^				
Yes	21 (35.2)	15 (20.2)	28 (44.5)	0.57 (0.27-1.20)
No	282 (44.0)	162 (25.8)	200 (30.1)	1 [Reference]
Technology access				
High	221 (39.3)	153 (27.1)	196 (33.6)	1 [Reference]
Moderate	37 (64.5)	5 (5.6)	16 (29.9)	2.65 (1.12-6.25)
Low	46 (60.5)	20 (24.1)	17 (15.3)	1.64 (0.74-3.65)
Use of internet-based video or voice calls^k^				
Yes	122 (28.5)	143 (33.5)	171 (38.0)	0.19 (0.11-0.33)
No	182 (67.8)	35 (11.8)	57 (20.4)	1 [Reference]

Abbreviations: FPL, federal poverty level; OR, odds ratio.

^a^ Unweighted numbers and weighted row percentages of responses to each survey item among respondents in group 3 are presented (Figure).

^b^ The multivariable model included all listed covariates. In the model, missing values were either categorized as no (for ethnicity, dementia, mental illness, hearing impairment, and vision impairment) or included using an indicator variable.

^c^ Other race includes American Indian or Alaska Native, Asian, Native Hawaiian or Pacific Islander, and more than 1 race. A total of 26 respondents answered “do not know” or refused to answer regarding race.

^d^ A total of 5 respondents answered “do not know” or refused to answer regarding ethnicity.

^e^ A total of 1 respondent answered “do not know” or refused to answer regarding limited English proficiency.

^f^ A total of 1 respondent answered “do not know” or refused to answer regarding dementia.

^g^ A total of 1 respondent answered “do not know” or refused to answer regarding mental illness.

^h^ A total of 2 respondents answered “do not know” or refused to answer regarding poor self-rated health.

^i^ A total of 2 respondents answered “do not know” or refused to answer regarding hearing impairment.

^j^ A total of 3 respondents answered “do not know” or refused to answer regarding vision impairment.

^k^ A total of 1 respondent answered “do not know” or refused to answer regarding use of internet-based video or voice calls.

## Discussion

In this study, Medicare beneficiaries commonly reported that clinicians offered them telephone visits or that beneficiaries chose telephone visits, even when both telephone and video visit options were reportedly available. These results suggest the need to expand the current policy debate beyond patients lacking video technology access, to consider the roles of practice availability, clinician offering, and patient preferences in the use of telephone visits. In light of these findings, telephone visits should remain available to patients to the extent that they facilitate access to care,^21^ and policymakers and clinicians should address both practice-level and patient-level barriers to video visit use over the longer term.

Our finding that 43.1% of patients chose telephone visits when given the option between telephone and video visits underscores the often-overlooked idea that patients may prefer telephone visits in some cases. Telephone visits may also be clinically reasonable for certain scenarios, such as in the treatment of mental health conditions.^22,23,24^ In further support of potential use cases for telephone visits, an analysis of video and telephone visits at 2 geriatrics clinics found that telephone visits were shorter than video yet included discussion of more diagnoses and were equally likely to include advance care planning.^17^

At the same time, given the likely advantages associated with video visits, we were concerned to find lower rates of video visits being offered and received among historically marginalized groups, consistent with prior studies finding fewer video visits among patients who are older,^7,8,9,10,11,12,13,14,15,16^ have lower incomes,^8^ are of Hispanic ethnicity,^8,9,16^ or have limited English proficiency.^7,9,13,17^ Although we found that Hispanic adults and those with limited English proficiency were substantially more likely to be offered only a telephone visit, they were not more likely to choose a telephone visit when given the option between telephone and video visits—in fact, they had lower odds of choosing a telephone visit, although these results were not statistically significant. These results suggest the possibility of implicit biases or assumptions on the part of clinicians or staff members offering visits (eg, if they believe that these patients may have a harder time navigating video technology). There may also be pragmatic concerns (for instance, it may be easier for a clinician to access interpreter services for telephone visits compared with video visits).

These findings also support the association of technology access with choice of modality. At each stage examined, patients without access to both internet and a smartphone or tablet were more likely to report being offered or receiving only telephone visits. Respondents who used video calls in other settings were consistently and markedly less likely to report telephone-only access or use. These findings support the possibility that, as use of video technology increases over time in settings outside of health care (eg, “FaceTime-ing” with grandchildren), adults may be more amenable to choosing video visits in health care settings if given the option.

We found an inverse association between dementia diagnosis and report of telephone-only offering—given the known underdiagnosis of dementia,^25^ this finding may reflect that more well-resourced practices are more likely both to diagnose dementia and to offer video visits. We also found that patients with lower educational levels were less likely to choose telephone visits, contrasting with results from an online US Census Bureau survey that was not specific to Medicare beneficiaries and found the opposite.^10^ Similar to another study,^17^ we did not note any associations between visit modality and vision or hearing impairment.

### Implications

Given the common use of telephone visits and that nearly half of Medicare beneficiaries reported choosing telephone visits when given the option, it may be important for policy makers to extend pandemic-era reimbursement of telephone visits as a modality that likely facilitates access to care in the short term and may be suitable in certain scenarios. At the same time, given the potential benefits associated with video visits, our results argue for the need to target practice-level, clinician-level, and patient-level barriers to video visit use over time. For example, to ensure that all practices offer video visits, new policies can offer financial support to practices for the needed infrastructure as well as regulatory incentives to motivate adoption of video visits. To encourage clinicians to offer video visits to all patients, payers might reimburse for telephone visits only if clinicians certify in their notes that they offered a video visit and the patient asked for a telephone visit instead—Medicare recently implemented this strategy for mental health telemedicine.^23^ In parallel, efforts to expand technology access are critical, especially those directed to key populations. For example, Silva et al^26^ described a culturally tailored program to engage Latinx patients in telehealth use. Finally, attention to user-friendly design is critical^5^ (eg, allowing clinicians to text video links to their patients to facilitate logging on to the visits). Future research should further clarify patient preferences, examine health and health care use outcomes linked to telephone vs video visits, and clarify optimal use cases for each modality.

### Limitations

This observational study does not allow causal inference and should be assessed in the context of its limitations. First, while our study is limited to use of virtual visits in the context of one’s usual source of care, most telehealth visits for older adults do take place within established clinician-patient relationships.^27^ Second, the survey was conducted earlier in the COVID-19 pandemic, and use of telephone visits may have evolved during the pandemic. Third, survey items are subject to recall bias and interpretation. For example, patients may inaccurately report what modalities their practice offers; responses indicating that one’s practice offered only telephone visits may in some cases reflect practice staff or clinicians withholding the video visit option for that respondent or patients with less technology access being less aware of the video visit option. Despite these limitations of patient report, it is important to understand patient perceptions of care access. Moreover, claims-based analysis of telephone vs video visits has its own substantial limitations.^28^ In addition, while these survey data are nationally representative, our results may not be generalizable to all Medicare beneficiaries, due to nonresponse. Fourth, in some cases, sample sizes may have been too small to establish statistical significance; in those cases, the effect estimates allow interpretation of the clinical significance of an association. Fifth, while these survey data allowed us to isolate patients’ actual (rather than hypothetical) choice of telephone visits when given both options and to use rich sociodemographic, clinical, and technology data to unpack factors that may explain this choice, we could not capture privacy preferences or other factors that may have helped to explain this choice.

## Conclusions

In this survey, Medicare beneficiaries reported that clinicians commonly offer, and that these beneficiaries commonly choose, telephone visits even when video visits are available. These results suggest the need to ensure access to telephone visits to the extent that these visits facilitate access to care in the short term while addressing practice-level, clinician-level, and patient-level barriers to video visit use in the longer term.

## Notes

Supplement 1. **eTable.** Nonresponse Analysis

Supplement 2.
Data Sharing Statement

## References

[1] Brodie M, Flournoy RE, Altman DE, Blendon RJ, Benson JM, Rosenbaum MD. Health information, the Internet, and the digital divide. Health Aff (Millwood). 2000;19(6):255-265. doi:10.1377/hlthaff.19.6.255 11192412

[2] Velazquez D, Mehrotra A. Ensuring the growth of telehealth during COVID-19 does not exacerbate disparities in care. Health Affairs. Published online May 8, 2020. Accessed October 6, 2022. https://www.healthaffairs.org/do/10.1377/forefront.20200505.591306/full/

[3] Gray J, Tengu D, Mehrotra A. 3 surprising trends in seniors’ telemedicine use during the pandemic. Stat News. Published August 30, 2021. Accessed November 12, 2021. https://www.statnews.com/2021/08/30/three-surprising-trends-seniors-telemedicine-use-pandemic/

[4] Samson LW, Tarazi W, Turrini G, Sheingold S. Medicare beneficiaries’ use of telehealth in 2020: trends by beneficiary characteristics and location. Office of the Assistant Secretary for Planning and Evaluation. Published December 3, 2021. Accessed December 8, 2021. https://aspe.hhs.gov/reports/medicare-beneficiaries-use-telehealth-2020

[5] Lam K, Lu AD, Shi Y, Covinsky KE. Assessing telemedicine unreadiness among older adults in the United States during the COVID-19 pandemic. JAMA Intern Med. 2020;180(10):1389-1391. doi:10.1001/jamainternmed.2020.2671 32744593 PMC7400189

[6] Rush KL, Howlett L, Munro A, Burton L. Videoconference compared to telephone in healthcare delivery: a systematic review. Int J Med Inform. 2018;118:44-53. doi:10.1016/j.ijmedinf.2018.07.007 30153920

[7] Chen J, Li KY, Andino J, . Predictors of audio-only versus video telehealth visits during the COVID-19 pandemic. J Gen Intern Med. 2022;37(5):1138-1144. doi:10.1007/s11606-021-07172-y 34791589 PMC8597874

[8] Eberly LA, Kallan MJ, Julien HM, . Patient characteristics associated with telemedicine access for primary and specialty ambulatory care during the COVID-19 pandemic. JAMA Netw Open. 2020;3(12):e2031640. doi:10.1001/jamanetworkopen.2020.31640 33372974 PMC7772717

[9] Rodriguez JA, Betancourt JR, Sequist TD, Ganguli I. Differences in the use of telephone and video telemedicine visits during the COVID-19 pandemic. Am J Manag Care. 2021;27(1):21-26. doi:10.37765/ajmc.2021.88573 33471458 PMC10877492

[10] Karimi M, Lee EC, Couture SJ, . National survey trends in telehealth use in 2021: disparities in utilization and audio vs. video services. Office of the Assistant Secretary for Planning and Evaluation. Accessed October 6, 2022. https://aspe.hhs.gov/reports/hps-analysis-telehealth-use-2021

[11] Pierce RP, Stevermer JJ. Disparities in the use of telehealth at the onset of the COVID-19 public health emergency. J Telemed Telecare. 2023;29(1):3-9. doi:10.1177/1357633X20963893 33081595 PMC7578842

[12] Gilson SF, Umscheid CA, Laiteerapong N, Ossey G, Nunes KJ, Shah SD. Growth of ambulatory virtual visits and differential use by patient sociodemographics at one urban academic medical center during the COVID-19 pandemic: retrospective analysis. JMIR Med Inform. 2020;8(12):e24544. doi:10.2196/24544 33191247 PMC7721629

[13] Sachs JW, Graven P, Gold JA, Kassakian SZ. Disparities in telephone and video telehealth engagement during the COVID-19 pandemic. JAMIA Open. 2021;4(3):ooab056. doi:10.1093/jamiaopen/ooab056 34632322 PMC8496485

[14] Drake C, Lian T, Cameron B, Medynskaya K, Bosworth HB, Shah K. Understanding Telemedicine’s “New Normal”: Variations in Telemedicine Use by Specialty Line and Patient Demographics. Telemed J E Health. 2022;28(1):51-59. doi:10.1089/tmj.2021.0041 33769092 PMC8785715

[15] Chen EM, Andoh JE, Nwanyanwu K. Socioeconomic and demographic disparities in the use of telemedicine for ophthalmic care during the COVID-19 pandemic. Ophthalmology. 2022;129(1):15-25. doi:10.1016/j.ophtha.2021.07.003 34245753 PMC8415734

[16] Eruchalu CN, Bergmark RW, Smink DS, . Demographic disparity in use of telemedicine for ambulatory general surgical consultation during the COVID-19 pandemic: analysis of the initial public health emergency and second phase periods. J Am Coll Surg. 2022;234(2):191-202. doi:10.1097/XCS.0000000000000030 35213441

[17] Schifeling CH, Shanbhag P, Johnson A, . Disparities in video and telephone visits among older adults during the COVID-19 pandemic: cross-sectional analysis. JMIR Aging. 2020;3(2):e23176. doi:10.2196/23176 33048821 PMC7674139

[18] Benjenk I, Franzini L, Roby D, Chen J. Disparities in audio-only telemedicine use among Medicare beneficiaries during the COVID-19 pandemic. Med Care. 2021;59(11):1014-1022. doi:10.1097/MLR.0000000000001631 34534186 PMC8516710

[19] Kurichi JE, Pezzin L, Streim JE, . Perceived barriers to healthcare and receipt of recommended medical care among elderly Medicare beneficiaries. Arch Gerontol Geriatr. 2017;72:45-51. doi:10.1016/j.archger.2017.05.007 28544946 PMC5522756

[20] Soumerai SB, Pierre-Jacques M, Zhang F, . Cost-related medication nonadherence among elderly and disabled Medicare beneficiaries: a national survey 1 year before the Medicare drug benefit. Arch Intern Med. 2006;166(17):1829-1835. doi:10.1001/archinte.166.17.1829 17000938

[21] Kyle MA, Tipirneni R, Thakore N, Dave S, Ganguli I. Primary care access during the COVID-19 pandemic: a simulated patient study. J Gen Intern Med. 2021;36(12):3766-3771. doi:10.1007/s11606-021-06804-7 33904036 PMC8075018

[22] Kim EK, Kidane J, Brodie S, Tuot DS, Sharon JD. Utility of telephone visits at an urban safety-net hospital during 2020: a retrospective review. Laryngoscope Investig Otolaryngol. 2022;7(5):1315-1321. doi:10.1002/lio2.875 36258874 PMC9575054

[23] Sweet K, Lacktman NM. CMS to permanently cover audio-only telemedicine for mental health. Foley & Lardner LLP. Published July 21, 2021. Accessed October 5, 2022. https://www.foley.com/en/insights/publications/2021/07/cms-cover-audio-only-telemedicine-mental-health

[24] Patel SY, Mehrotra A, Huskamp HA, Uscher-Pines L, Ganguli I, Barnett ML. Variation in telemedicine use and outpatient care during the COVID-19 pandemic in the United States. Health Aff (Millwood). 2021;40(2):349-358. doi:10.1377/hlthaff.2020.01786 33523745 PMC7967498

[25] Amjad H, Roth DL, Sheehan OC, Lyketsos CG, Wolff JL, Samus QM. Underdiagnosis of dementia: an observational study of patterns in diagnosis and awareness in US older adults. J Gen Intern Med. 2018;33(7):1131-1138. doi:10.1007/s11606-018-4377-y 29508259 PMC6025653

[26] Silva MA, Perez OFR, Añez LM, Paris M Jr. Telehealth treatment engagement with Latinx populations during the COVID-19 pandemic. Lancet Psychiatry. 2021;8(3):176-178. doi:10.1016/S2215-0366(20)30419-3 33038976 PMC7544483

[27] Office of Inspector General. Most Medicare beneficiaries received telehealth serices only from providers with whom they had an established relationship. Published October 2021. Accessed November 12, 2021. https://oig.hhs.gov/oei/reports/OEI-02-20-00521.pdf

[28] Hailu R, Uscher-Pines L, Ganguli I, Huskamp HA, Mehrotra A. Audio-only telemedicine visits: flaws in the underlying data make it hard to assess their use and impact. Health Affairs. Published July 15, 2022. Accessed August 2, 2022. https://www.healthaffairs.org/do/10.1377/forefront.20220713.689213

