# Supplementary material for: Patient Characteristics Associated With Being Offered or Choosing Telephone vs Video Virtual Visits Among Medicare Beneficiaries
Source: JAMA Netw Open. 2023 Mar 29;6(3):e235242. doi: 10.1001/jamanetworkopen.2023.5242 (PMC10061240; doi:10.1001/jamanetworkopen.2023.5242)
Supplement: Supplement 1. — eTable. Nonresponse Analysis [file jamanetwopen-e235242-s001.pdf]

Supplementary Online Content

Ganguli I, Orav EJ, Hailu R, et al. Patient characteristics associated with being offered or choosing telephone vs video virtual visits among Medicare beneficiaries. *JAMA Netw Open.* 2023;6(3):e235242. doi:10.1001/jamanetworkopen.2023.5242

eTable. Nonresponse Analysis

This supplementary material has been provided by the authors to give readers additional information about their work.

© 2023 Ganguli I et al. *JAMA Network Open.*

eTable. Nonresponse Analysis

	Primary Outcome 1			Primary Outcome 2		
	**Non-respondents N=749**	**Respondents N=4691**	**Standardized mean difference**	**Non-respondents N=26**	**Respondents N=1593**	**Standardized mean difference**
**Age**						
18-64	82(10.95)	770(16.41)	.159	6(23.08)	296(18.58)	.111
65-74	281(37.52)	1767(37.67)	.003	9(34.62)	629(39.49)	.101
75-84	265(35.38)	1561(33.28)	.044	5(19.23)	509(31.95)	.295
85+	121(16.15)	593(12.64)	.100	6(23.08)	159(9.98)	.358
**Sex**						
Male	305(40.72)	2116(45.11)	.089	12(46.15)	733(46.01)	.003
Female	444(59.28)	2575(54.89)	.089	14(53.85)	860(53.99)	.003
**Race**						
Black	40(5.34)	473(10.08)	.178	0(0)	156(9.79)	.466
White	667(89.05)	3818(81.39)	.217	24(92.31)	1280(80.35)	.354
Other	42(5.61)	400(8.53)	.114	2(7.69)	157(9.86)	.077
**Hispanic Ethnicity**	41(5.47)	557(11.87)	.229	3(11.54)	170(10.67)	.028
**Education**						
Did not graduate high school	74(9.88)	692(14.75)	.149	2(7.69)	197(12.37)	.156
High school/some college	353(47.13)	2241(47.77)	.013	15(57.69)	765(48.02)	.195
College or above	322(42.99)	1758(37.48)	.113	9(34.62)	631(39.61)	.103
**Income**						
<=100% FPL	86(11.48)	780(16.63)	.149	3(11.54)	224(14.06)	.075
100%-≤200% FPL	183(24.43)	1190(25.37)	.022	5(19.23)	424(26.62)	.176
>200% FPL	480(64.09)	2721(58.00)	.125	18(69.23)	945(59.32)	.208
**Limited English Proficiency**	13(1.74)	333(7.10)	.263	0(0)	75(4.71)	.314
**House type**						
Apt/Condo	106(14.15)	812(17.31)	.087	4(15.38)	273(17.14)	.048
Trailer	51(6.81)	298(6.35)	.019	0(0)	99(6.21)	.364
House	592(79.04)	3581(76.34)	.065	22(84.62)	1221(76.65)	.203

We did not report a nonresponse analysis for the third primary outcome as there were only 3 non-respondents and Centers for Medicare and Medicaid Services data reporting rules require suppression of cells with <11 beneficiaries.

© 2023 Ganguli I et al. *JAMA Network Open.*

eTable 1. Nonresponse Analysis (cont.)

	Primary Outcome 1			Primary Outcome 2		
	Non-respondents N=749	Respondents N=4691	Standardized mean difference	Non-respondents N=26	Respondents N=1593	Standardized mean difference
**Number in home**						
1	249(33.24)	1278(27.24)	.131	5(19.23)	425(26.68)	.178
2	389(51.94)	2366(50.44)	.030	16(61.54)	804(50.47)	.224
3+	111(14.82)	1047(22.32)	.194	5(19.23)	364(22.85)	.089
**Dementia**	22(2.94)	188(4.01)	.058	0(0)	61(3.83)	.282
**Mental Illness**	183(24.43)	1459(31.10)	.149	8(30.77)	566(35.53)	.101
**Poor Self-Health**	120(16.02)	1041(22.19)	.157	7(26.92)	381(23.92)	.069
**Hearing Impaired**	130(17.36)	788(16.80)	.015	8(30.77)	269(16.89)	.330
**Vision Impaired**	62(8.28)	400(8.53)	.009	3(11.54)	131(8.22)	.111
**Technology Access**						
High	525(70.09)	3302(70.39)	.007	19(73.08)	1220(76.59)	.081
Moderate	92(12.28)	549(11.70)	.018	3(11.54)	166(10.42)	.036
Low	132(17.62)	840(17.91)	.008	4(15.38)	207(12.99)	.069
**Uses internet-based video or voice calls**	308(41.12)	2111(45.00)	.078	13(50.00)	877(55.05)	.101

We did not report a nonresponse analysis for the third primary outcome as there were only 3 non-respondents and Centers for Medicare and Medicaid Services data reporting rules require suppression of cells with <11 beneficiaries.

© 2023 Ganguli I et al. *JAMA Network Open.*
