# Supplementary material for: Patient Characteristics Associated With Being Offered or Choosing Telephone vs Video Virtual Visits Among Medicare Beneficiaries
Source: JAMA Netw Open. 2023 Mar 29;6(3):e235242. doi: 10.1001/jamanetworkopen.2023.5242 (PMC10061240; doi:10.1001/jamanetworkopen.2023.5242)
Supplement: Supplement 2. — Data Sharing Statement [file jamanetwopen-e235242-s002.pdf]

Data Sharing Statement

Ganguli. Patient Characteristics Associated With Being Offered or Choosing Telephone vs Video Virtual Visits Among Medicare Beneficiaries. *JAMA Netw Open*. Published March 29, 2023. doi:10.1001/jamanetworkopen.2023.5242

Data
Data available: No

Additional Information
Explanation for why data not available: These data were purchased from, and are subject to a data use agreement with, the Centers for Medicare and Medicaid Services.
